# Functional Characterization of Dense Granule Proteins in *Toxoplasma gondii* RH Strain Using CRISPR-Cas9 System

**DOI:** 10.3389/fcimb.2018.00300

**Published:** 2018-08-28

**Authors:** Meng-Jie Bai, Jin-Lei Wang, Hany M. Elsheikha, Qin-Li Liang, Kai Chen, Lan-Bi Nie, Xing-Quan Zhu

**Affiliations:** ^1^State Key Laboratory of Veterinary Etiological Biology, Key Laboratory of Veterinary Parasitology of Gansu Province, Lanzhou Veterinary Research Institute, Chinese Academy of Agricultural Sciences, Lanzhou, China; ^2^Faculty of Medicine and Health Sciences, School of Veterinary Medicine and Science, University of Nottingham, Loughborough, United Kingdom; ^3^College of Animal Science and Technology, Jilin Agricultural University, Changchun, China

**Keywords:** *Toxoplasma gondii*, CRISPR-Cas9, dense granule proteins (GRAs), host-pathogen interaction, virulence

## Abstract

Infection with the apicomplexan protozoan parasite *Toxoplasma gondii* is an ongoing public health problem. The parasite's ability to invade and replicate within the host cell is dependent on many effectors, such as dense granule proteins (GRAs) released from the specialized organelle dense granules, into host cells. GRAs have emerged as important determinants of *T. gondii* pathogenesis. However, the functions of some GRAs remain undefined. In this study, we used CRISPR-Cas9 technique to disrupt 17 *GRA* genes (*GRA11, GRA12 bis, GRA13, GRA14, GRA20, GRA21, GRA28-31, GRA33-38*, and *GRA40*) in the virulent *T. gondii* RH strain. The CRISPR-Cas9 constructs abolished the expression of the 17 *GRA* genes. Functional characterization of single Δ*GRA* mutants was achieved *in vitro* using cell-based plaque assay and egress assay, and *in vivo* in BALB/c mice. Targeted deletion of these 17 *GRA* genes had no significant effect neither on the *in vitro* growth and egress of the mutant strains from the host cells nor on the parasite virulence in the mouse model of infection. Comparative analysis of the transcriptomics data of the 17 *GRA* genes suggest that *GRAs* may serve different functions in different genotypes and life cycle stages of the parasite. In sum, although these 17 GRAs might not be essential for RH strain growth *in vitro* or virulence in mice, they may have roles in other strains or parasite stages, which warrants further investigations.

## Introduction

*Toxoplasma gondii* is an obligate intracellular apicomplexan protozoan parasite that can infect all warm-blooded animals, mainly through oral and congenital infections (Tenter et al., 2000; Elmore et al., 2010; Zhou et al., 2011). This parasite has a significant medical and socioeconomic importance because it infects over two billion people worldwide. It is the causative agent of toxoplasmosis, an important zoonotic disease that can cause serious, even fatal health consequences in immunocompromised individuals, such as AIDS patients and organ transplant recipients (Liu et al., 2008; Xiao et al., 2010; Chemoh et al., 2015). Infection in immunocompetent individuals are generally asymptomatic, however the parasite persists as bradyzoites-containing cysts in brain and muscle tissues for many years. Primary infection in pregnant women can lead to fetus death, deformity, abortion, and long-term damage of the eye and central nervous system (Hill and Dubey, 2002).

The remarkable ability of *T. gondii* to invade and colonize virtually all nucleated cells (Morisaki et al., 1995), and to adopt a successful intracellular lifestyle depends partly on the sequential secretion of effectors from the specialized secretory organelles micronemes, rhoptries and dense granules (Håkansson et al., 2001; Boothroyd and Dubremetz, 2008; Nadipuram et al., 2016). *T. gondii* tachyzoites enter the host cells through an active invasion mechanism and replicate within a membrane-bound parasitophorous vacuole (PV) inside the surrogate host cell cytoplasm (Coppens et al., 2006; Nam, 2009). After release from the host cell, newly formed parasites invade new mammalian host cells, and the replication cycle starts again. Most of the dense granule proteins (GRAs) are destined to the PV and parasitophorous vacuole membrane (PVM), and contribute to the biogenesis and maturation of the PV, and nutrient acquisition (Mercier and Cesbron-Delauw, 2015).

Some GRA proteins have the ability to traffic to the host cytoplasm or nucleus and interfere with host cell signaling pathways. For example, *GRA6* is a polymorphic dense granule protein and activates the host transcription nuclear factor of activated T cells 4 (NFAT4) in order to manipulate host immune responses to maximize the parasite virulence in a strain-specific manner (Ma et al., 2014). *GRA15* is another strain-specific effector that activates NF-κB pathway and induces IL-12 secretion in type II, but not type I or III genotypes. *GRA15*-deficient type II strain cannot activate NF-κB pathway or induces IL-12 secretion, hence *GRA15*-deficient type II strains grow faster compared with wild-type strains (Rosowski et al., 2011). *GRA16* and *GRA24* also manipulate host gene expression and signaling pathways (Bougdour et al., 2013; Braun et al., 2013). *GRA17* mediates the transfer of small molecules between the host cell and PV, and maintains the stability of the PV (Gold et al., 2015). *GRA7, GRA25*, and *GRA39* are also virulence factor and can interfere with host cell signaling pathways (Alaganan et al., 2014; Shastri et al., 2014; Nadipuram et al., 2016). *GRA22* and *GRA41* are involved in the parasite egress (Okada et al., 2013; LaFavers et al., 2017).

Despite the wealth of information regarding *T. gondii*'s 40+ GRAs including sequence variation and expression of GRA coding genes, and their roles in the infection process, the contribution of many GRAs to the parasite growth and virulence are still unclear. The CRISPR-Cas9 system provides a novel and promising tool for editing *T. gondii* genes (Shen et al., 2014) and the genome (Sidik et al., 2014; Wang et al., 2016a; Shen et al., 2017). Using CRISPR-Cas9 to target *T. gondii*'s GRA genes may ultimately offer a new approach to achieving functional cure to toxoplasmosis. In this study, we used the CRISPR-Cas9 technique to edit 17 GRA genes, namely *GRA11, GRA12 bis, GRA13, GRA14, GRA20, GRA21, GRA28-31, GRA33-38*, and *GRA40* in the virulent *T. gondii* RH strain and examined the effects of gene loss on the parasite's ability to grow and exit from host cells *in vitro* and to cause death in mice.

## Materials and methods

### Parasite and cell cultures

Tachyzoites of *T. gondii* RH strain (type I) were maintained *in vitro* by passages in human foreskin fibroblast (HFF, ATCC, Manassas, VA, USA). HFF cells were grown in 75-cm^2^ tissue culture flasks containing Dulbecco's Modified Eagle medium (DMEM) supplemented with 10% fetal bovine serum (FBS) and 10 μg/ml gentamycin, at 37°C under a 5% CO_2_ atmosphere. To purify tachyzoites, infected HFF cells were lysed through a 27-gauge needle and the tachyzoites were filtered using a 5 μm pore size Millipore filter. The number of purified parasites was counted using a hemocytometer under a phase-contrast microscopy.

### Construction of GRA knockout *T. gondii* strains using CRISPR-Cas9

*GRA* knockout strains were constructed by using CRISPR-Cas9 as previously described (Wang et al., 2016b). All plasmids, primers, and gRNAs used in this study are listed in Table S1. Briefly, sgRNA of each *GRA* was engineered into pSAG1::CAS9-U6::sgUPRT by PCR using the Q5 Mutagenesis Kit (NEB). Positive plasmid was extracted with Endo-Free Plasmid DNA Mini Kit Protocols (OMEGA). The resistance cassettes (DHFR^*^-Ts) were amplified from the plasmid pUPRT-DHFR-D and purified by agarose gel electrophoresis. About 40 μg positive plasmids and 15 μg purified DHFR^*^-Ts amplicons were co-transfected into freshly harvested *T. gondii* RH tachyzoites by electroporation. *GRA*-deficient tachyzoites were selected with pyrimethamine and examined by PCR analysis. Stable clones were confirmed by reverse transcription PCR (RT-PCR) by comparison to WT strains. Total RNA was extracted from tachyzoites of wild type (WT) or Δ*GRA* mutant *T. gondii* strains using TRIzol (Invitrogen). Reverse transcription was performed using a PrimeScriptTM 1st Strand cDNA Synthesis Kit (TaKaRa). The central region of each target gene cDNA was amplified by RT-PCR using specific primers (see Table S1 in the Supplemental Material).

### Assessment of parasite growth using plaque assay

The growth rates of individual *GRA*-deficient and WT RH strains were determined in HFF cells. Cells were grown to confluence overnight in 6-well cell culture plates. The cells were then infected with tachyzoites of Δ*GRA* mutant and WT RH strains (~200 tachyzoites/well). Cells were incubated for 3 h to allow the parasites to enter the host cells and were then washed twice with sterile 1 × phosphate-buffered saline (PBS) to remove unbound parasites, and fresh medium was added. The plates were incubated for 7 days at 37°C in 5% CO_2_ environment. Then, the culture medium was removed and infected HFF cells were fixed with 4% paraformaldehyde in PBS (pH 7.4) for 15 min at ambient temperature. Then, fixed cells were incubated with crystal violet staining solution (2% [wt/vol] crystal violet, pH 7.4) for 10 min at ambient temperature. The size of plaques (i.e., areas in the cell culture devoid of cells caused by the proliferating parasites) was determined using inverted microscope as previously described (Wang et al., 2017). This experiment was performed in triplicate and the experiments were repeated three independent times.

### Egress assay

Asexual reproduction of *T. gondii* culminates with the egress of the newly formed tachyzoites from the surrogate host cell and subsequent parasite invasion into new host cells. Therefore, parasite egress represents a very important event which is indispensable for the parasite dissemination in the host body. In order to determine the role of the GRAs in this important process, we examined the effect of ionophore A23187 modulating Ca2^+^ homeostasis on the egress of the parasite from the host cells. Briefly, HFF cells were incubated with 10^5^ freshly harvested *T. gondii* tachyzoites in 2 ml culture medium for 3 h, followed by washing twice with DMEM medium to remove the unbound parasites. After 30–36 h of incubation, the wells were washed twice with sterile PBS and 3 μM calcium ionophore A23187 (Sigma) diluted in DMSO were added to the HFF cells. Live cell microscopy was used to monitor the timing of parasite egress from HFF cells infected with the WT strain compared with HFF cells infected with the mutant strains after addition of 3 μM calcium ionophore A23187.

### Mouse infection with *GRA*-deficient strains

Specific-pathogen-free (SPF) inbred female BALB/c mice (8 weeks-old) were purchased from Center of Laboratory Animals, Lanzhou Veterinary Research Institute, Lanzhou, China. Mice (5 mice/cage) were housed in a SPF environment within the animal care facility during the experiment. Each mouse was injected intraperitoneally with 200 freshly harvested tachyzoites of *GRA*-deficient or WT RH strain (10 mice per strain). The negative control mice were injected with an equal amount PBS only. All mice were monitored daily for the signs of illness and time of death.

### Bioinformatics analysis of *T. gondii* dense granule proteins

Information on the genomic features (signal peptide, the number of exons and transmembrane domains) and time-series expression data of the *GRA* genes by the parasite cell cycle phases, parasite life cycle stages (the oocyst, tachyzoite, and bradyzoites), and the parasite genotypes were downloaded from ToxoDB (http://ToxoDB.org; Gajria et al., 2007). *GRA* gene expression data were processed using Robust Multiarray Average (RMA) algorithm of the Partek Genomics Suite package (Partek, Inc, St Louis, MO, USA).

### Statistical analysis

Statistical analysis for the *in vitro* and *in vivo* experiments was carried out using Graphpad Software package (GraphPad Software, La Jolla, CA). All experiments in the present study were conducted with at least 3 replicate and data were shown as means ± standard deviations (SD). Significant differences between means were determined by Student's *t*-test. *P*-values below 0.05 were considered significant.

## Results and discussion

In this study, we assessed the impact of targeted disruption of the individual 17 *GRA* genes (*GRA11, GRA12 bis, GRA13, GRA14, GRA20, GRA21, GRA28-31, GRA33-38*, and *GRA40*) on the ability of the virulent *T. gondii* RH tachyzoites to grow and exit from the cultured HFF cells. We also examined the impact of the disruption of *GRA* genes on the virulence of *T. gondii* in BALB/c mice.

### Generation of single Δ*GRA* knockout RH strains

**Figure 1:**
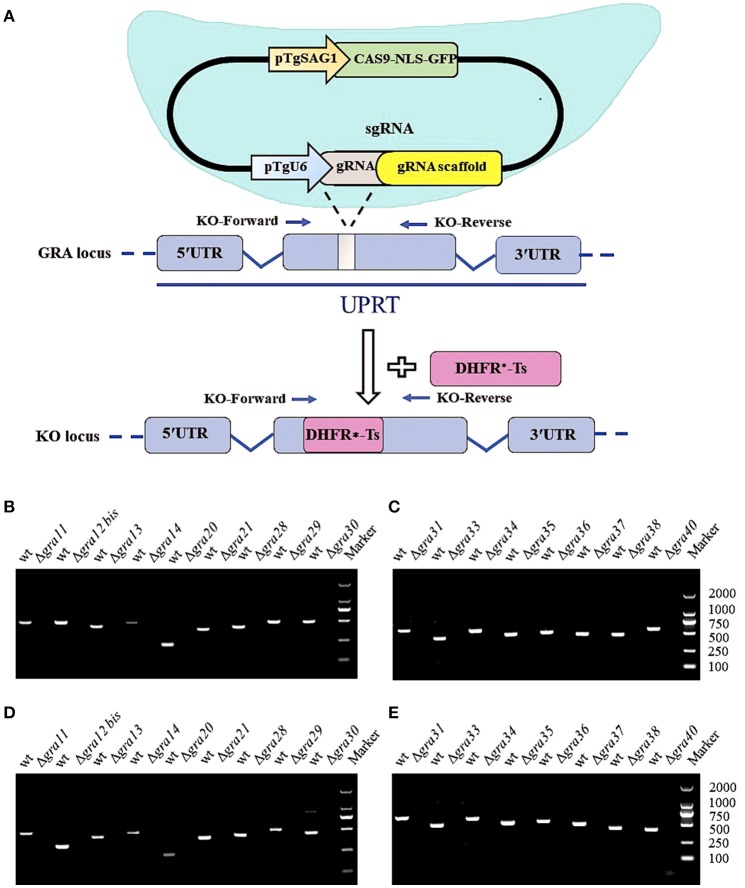
Overview of the CRISPR/Cas9 system and mutation analysis of Toxoplasma gondii dense granule proteins (GRA) genes**. (A)** Schematic representation of CRISPR-Cas9 system used for disrupting the 17 *GRAs* genes by insertion of DHFR^*^-Ts cassette. **(B,C)** Diagnostic PCR shows *GRA* gene disruption in the mutants compared to the wild-type strain. The KO-forward and KO-reverse primers were used to amplify the small fragment with 30 s extension time. **(D,E)** RT-PCR of mRNA from parental RH (WT) and *GRA*-deficient strains, showing that the 17 GRA's coding sequences were successfully disrupted. Marker denotes the DNA ladder.

CRISPR-Cas9 technique was used to disrupt the *GRA* genes in type I RH strain. We designed RNA-guided CRISPR-Cas9 targeting *T. gondii* 17 *GRA* genes individually. The larger pyrimethamine-resist fragment (DHFR^*^-Ts) was designed for inserting into the sgRNA-targeted coding region causing frameshift mutation for GRA proteins (Figure 1A). Then, single clones were identified by PCR, and small fragment (~500 bp) was not amplified in *GRA*-deficient strains due to the insertion of the large DHFR^*^-Ts fragment with short extension time, however it was detected in the WT strain (Figures 1B,C). RT-PCR was used to amplify cDNA products, reverse transcribed from mRNA, of each *GRA* gene. Our result showed that CRISPR-Cas9 abolished the expression of *GRA* genes in the transfected strains compared with the WT strains (Figures 1D,E), indicating that the target *GRA* genes were mutated at the Cas9 cleavage sites and that 17 Δ*GRA* mutant strains were successfully generated.

### Deletion of *GRA* genes did not affect the parasite growth and egress

**Figure 2:**
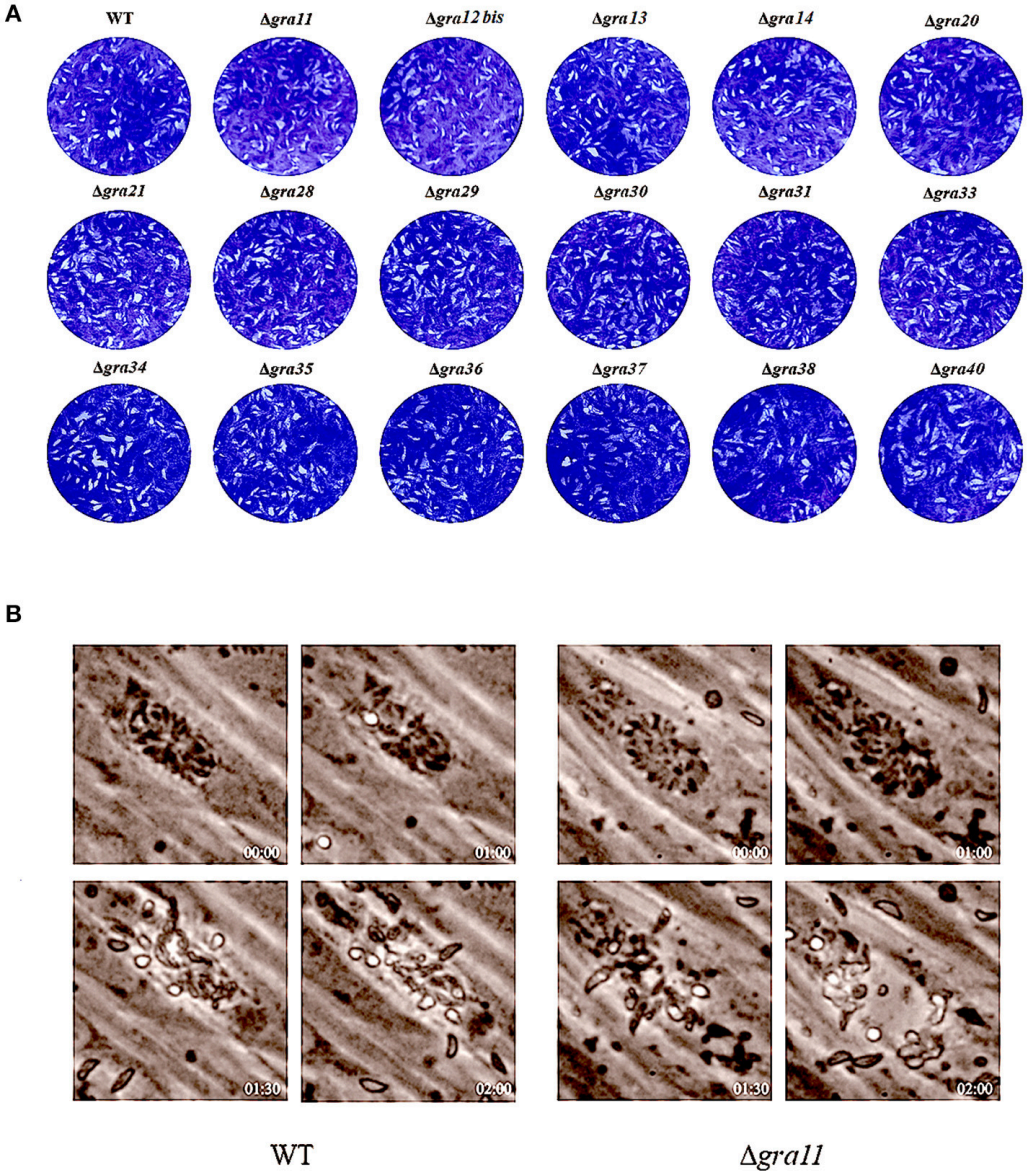
Phenotypic characterization of *GRA* Knockouts *in vitro*. **(A)** Two hundred freshly harvested *T. gondii* tachyzoites of WT RH strains and *GRA*-deficient RH strains per well were added to monolayers of HFF cells in 6-well culture plates. After 7 days, the number of plaques caused by the parasite's proliferation was counted using a microscope. No differences were detected in the number of plaques produced by wild-type (WT) RH strain *vs*. any of the *GRA* knockout strains. **(B)** Representative images show the parasite egress of the parental WT RH strain and one of the *GRA* mutant RH strains (Δ*gra11*). Live cell imaging showed a similar egress pattern between WT and all mutant parasites after addition of 3 μM calcium ionophore A23187 at the indicated time points.

After confirming knockout by RT-PCR, we compared the plaque formation in HFF monolayers grown in 6-well culture plates 7 days after infection with 200 tachyzoites of wild-type and Δ*GRA* mutant strains by conventional crystal violet staining and microscopic examination. As shown in Figure 2A, no significant differences in the size of plaques were observed in cells infected with wild-type compared to cells infected with Δ*GRA* mutant strains (*p* = 0.1582). Next, we evaluated the role of *GRAs* in the parasite egress. The infected HFF cells were treated with 3 μM calcium ionophore A23187 and the timing of the parasite exit from the cells was monitored over 5 min (Figure 2B). No significant difference in the parasites egress was observed between Δ*GRA* mutants and WT strains. The 17 *GRA* KO strains and the WT strains remained within the PV after stimulation with DMSO. Calcium is a critical mediator of *T. gondii* invasion and egress processes. The calcium ionophore A23187 can stimulate the parasite to exit from the PV to the extracellular space (Arrizabalaga et al., 2004; Caldas et al., 2010). *GRA41* can regulate the timing of egress and the sensitivity to calcium (LaFavers et al., 2017). However, none of the 17 *GRA* mutant strains tested in the present study seem to be responsive to the action of the A23187. In agreement with our results, a previous study has shown that deletion of 15 rhoptry organelle proteins ROPs (*ROP10, ROP11, ROP15, ROP20, ROP23, ROP31, ROP32, ROP33, ROP34, ROP35, ROP36, ROP40, ROP41, ROP46*, and *ROP47*) did not suppress the parasite's ability to grow in HFF cells or alter its pathogenicity for BALB/c mice (Wang et al., 2017). This lack of effect of *GRA* deletion on the phenotype of single parasite mutants argues for possible redundancy of function for *GRAs*.

### *GRA* deletion did not alter the parasite virulence in mice

**Figure 3:**
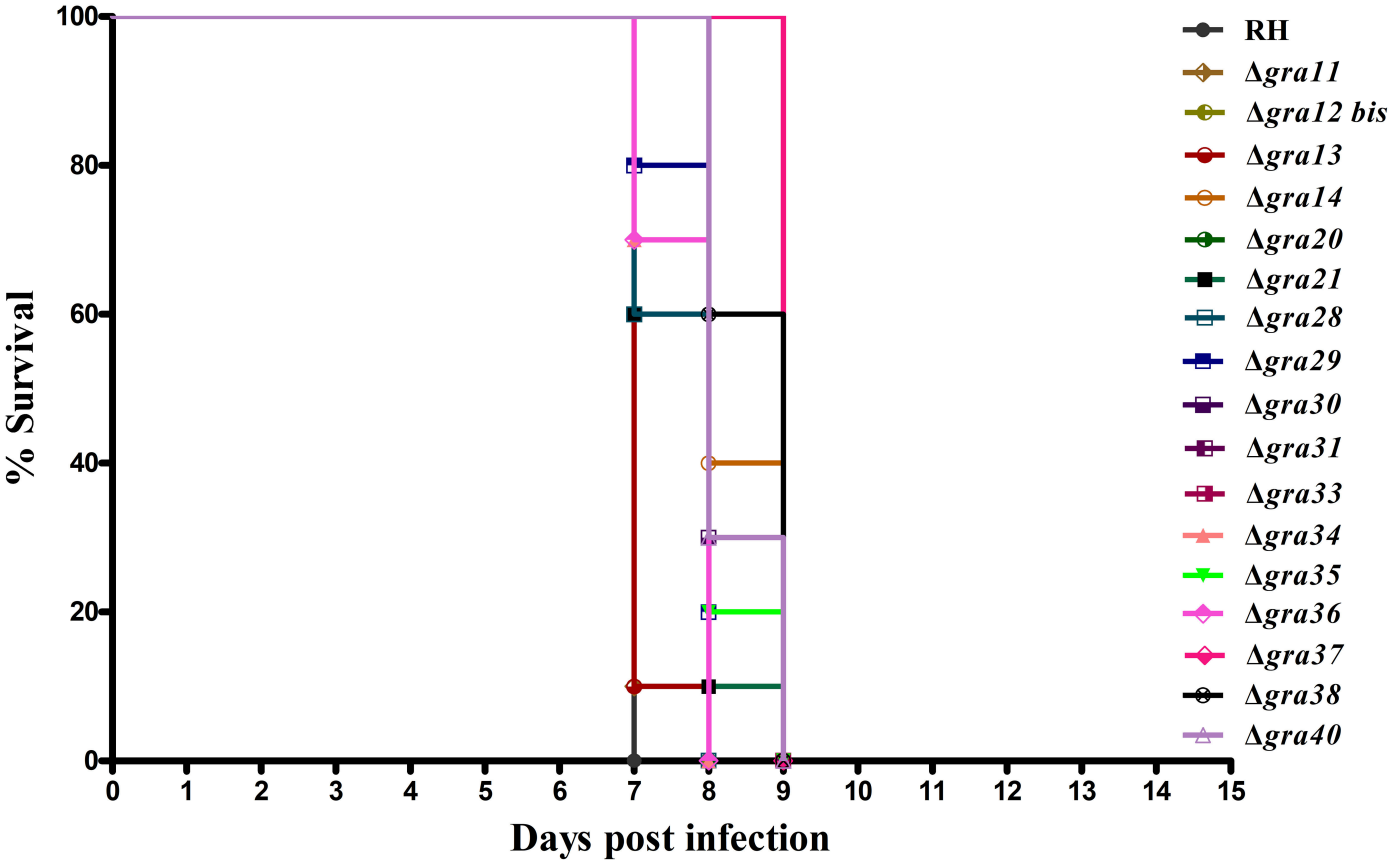
Survival of BALB/c mice infected with *Toxoplasma gondii* wild-type or *GRA*-deficient RH strains. The mice were injected i.p., with 200 freshly harvested tachyzoites of the indicated strains. Ten mice were used per parasite strain. The survival time was recorded daily until all the mice have died within 7–9 days post challenge.

We tested whether deletion of *GRA* genes in *T. gondii* RH strains would cause any changes in the parasite virulence. We inoculated female BALB/c mice intraperitoneally with 200 *T. gondii* tachyzoites (wild-type or mutants) and monitored the mortality and signs of illness on a daily basis. All mice died within 7–9 days (Figure 3), suggesting that the 17 *GRA* genes tested in the present study might not contribute to the parasite virulence during infection. Previous studies have shown that some of dense granule proteins such as *GRA25* may regulate the production of CCL2 and CCL1 in macrophages and thus affect virulence, which was different between Type II and Type III strains (Shastri et al., 2014). *GRA39* is also an important virulence factor in Type II strains (Nadipuram et al., 2016). However, our results did not show any significant differences between WT and Δ*GRA* mutant strains. The fact that Δ*GRA* strains did not display any reduction or loss of pathogenicity in mice suggests that deletion of a single gene might not be enough to influence the highly virulent RH strain in which the knockout was performed. More research is required to elucidate the impact of individual or multiple *GRA* gene deletion on the phenotype and virulence of *T. gondii* strains. It is possible that these *GRA* genes have roles in the pathogenesis of *T. gondii*, but in *T. gondii* strains of other genotypes or in other hosts. Disruption of these GRAs in avirulent and more physiologically relevant cystogenic strains may allow the assessment of more subtle roles in the virulence, alteration in cyst formation or tissue tropism.

### Sequence and expression analyses of *GRAs* in *T. gondii*

**Figure 4:**
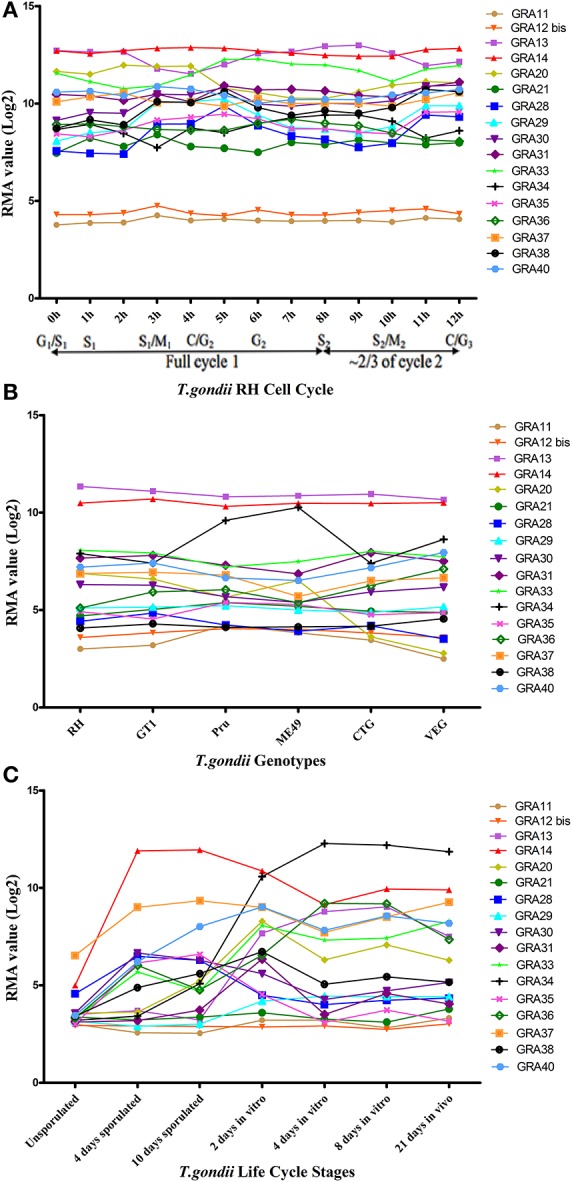
The trend charts of the distinct expression profiles of *Toxoplasma gondii GRAs***. (A)** Time-series expression profile of 17 *GRA* genes of *T. gondii* RH strain by cell cycle phases of the parasite as described by Behnke et al. (2010). **(B)** Transcriptomic expression profiles of 17 *GRA* genes in Type I (RH and GT1), Type II (Pru and ME49), and Type III (CTG and VEG) strains. **(C)** Transcriptomic profiles of 17 *GRA* genes related to the parasite life cycle stages (oocyst, tachyzoite and bradyzoite). Expression profile of 17 *GRA* genes of the oocysts recovered from cat feces, at 0 day (unsporulated), 4 days (4 day sporulated), and 10 days (10 day sporulated), tachyzoites grown for 2 days in HFF cells (2 day *in vitro*), bradyzoites grown in HFF cells for 4 days and 8 days (4 day *in vitro* and 8 day *in vitro*), and 21 days tissue cyst-containing bradyzoites harvested from infected mouse brains (21 day *in vivo*). Each line represents the expression value of the corresponding gene. The data were obtained from ToxoDB (36 release) and the graph was generated using GraphPad Prism version 5.0.

More than one-third of *T. gondii* mRNAs exhibit a tightly regulated expression pattern, probably driven by the diverse cell-cycle-dependent processes mediated by the parasite in the course of infection (Behnke et al., 2010). We mapped the transcriptomic data of the 17 *GRAs* available in ToxoDB and found that the expression profile of the majority of *GRAs* do not follow a particular cell cycle pattern, and that *GRA11* and *GRA12 bis* expression level was low (Figure 4A). Bioinformatics features of *GRAs*, such as the number of exons, signal peptide and transmembrane domains are summarized in Table 1. The majority of the known *GRAs* include a signal peptide at the N terminus and a single transmembrane domain in the C-terminal part of the protein. We found that some GRAs encode multi-exons (even eight) and a small amount of *GRAs* do not have a signal peptide or a transmembrane domain. The signal peptide plays an important role in protein targeting and protein translocation in and eukaryotic cells and is considered as a feature of the GRA proteins that enter the secretory pathway (Hakimi and Bougdour, 2015). Most of the previously identified GRA proteins have been predicted to contain classical or non-classical signal peptide, such as *GRA1, GRA2, GRA6, GRA9, GRA16*, and *GRA21*, whereas other GRAs (*GRA10, GRA15, GRA20*, and *GRA22*) did not (Mercier and Cesbron-Delauw, 2015). However, a few GRAs do not seem to depend on signal peptide to enter the secretory pathway, such as *GRA5*, which is secreted into the PV as a unique soluble protein and then binds to the PVM (Gendrin et al., 2008). Some GRA proteins lack the transmembrane domain, typically *GRA19, GRA20*, and *GRA21*, and may interact with the PVM through protein-protein interactions (Hsiao et al., 2013). Other GRAs (38, 39, and 40) lack the transmembrane domains or other identifiable sequences for membrane association, suggesting they are soluble protein of the PV (Nadipuram et al., 2016). It is possible that GRAs that lack signal peptide or transmembrane domain perform their roles by as yet unknown mechanisms.

**Table 1:** Bioinformatics features of GRA proteins of *Toxoplasma gondii*.

**Name**	**Gene ID**	**Product description**	**Exons**	**Phenotype value**	**TMHMM^a^**	**Predicted signal peptide**	**Acute infection ^b^**	**Chronic infection ^b^**
GRA11	TGGT1_212410	Dense granule protein GRA11	1	ND	Yes	Yes	5.97	5.21
GRA12 bis	TGGT1_275850	Dense granule protein GRA12	3	1.31	No	Yes	3.00	2.63
GRA13	TGGT1_237880	Hypothetical protein	1	0.76	Yes	Yes	120.50	34.28
GRA14	TGGT1_239740	Dense granule protein GRA14	1	2.00	No	Yes	116.36	53.11
GRA20	TGGT1_200010	Hypothetical protein	2	2.54	No	No	37.97	30.74
GRA21	TGGT1_241610	Hypothetical protein	2	ND	No	Yes	18.40	13.56
GRA28	TGGT1_231960	Putative omega secalin	3	1.48	No	Yes	33.28	3.58
GRA29	TGGT1_269690	Hypothetical protein	1	1.54	Yes	Yes	47.08	77.60
GRA30	TGGT1_232000	Hypothetical protein	2	2.29	Yes	Yes	33.46	44.61
GRA31	TGGT1_220240	Hypothetical protein	1	0.90	Yes	Yes	33.92	22.57
GRA33	TGGT1_247440	Hypothetical protein	1	1.72	Yes	Yes	48.06	45.42
GRA34	TGGT1_203290	Hypothetical protein	1	2.26	No	Yes	64.29	248.97
GRA35	TGGT1_226380	Hypothetical protein	1	1.98	Yes	Yes	48.74	32.29
GRA36	TGGT1_213067	Hypothetical protein	1	−0.21	Yes	Yes	83.85	24.24
GRA37	TGGT1_236890	Hypothetical protein	2	2.10	Yes	No	60.01	34.21
GRA38	TGGT1_312420	Hypothetical protein	4	−1.15	No	Yes	27.17	17.82
GRA40	TGGT1_219810	Hypothetical protein	7	0.69	Yes	No	114.88	97.16

*ND, Not determined*.

a *Prediction of transmembrane helices was performed using the TMHMM program version 2.0*.

b *Gene expression levels of fragments per kilobase of exon model per million mapped reads (FPKM) at acute (10 days post infection) and chronic infection (28 days post infection). Source: http://www.toxodb.org/toxo/ [accessed 26 July 2018]*.

We next analyzed the transcriptomic levels of 17 GRAs in different *T. gondii* genotypes (Type I, II, and III), and found that *GRA20* and *GRA34* were significantly different among the three genotypes (Figure 4B). This difference may be because these two GRAs play different roles in different strains. Levels of the transcriptomic expression of 17 *GRA* genes across *T. gondii* developmental stages are presented (Figure 4C). Our analysis showed that most GRAs are differentially expressed at different life cycle stages, but *GRA11* and *GRA21*. The expression of some *GRA* genes can be specifically up- or down-regulated during parasite development (Fritz et al., 2012). For instance, the expression of *GRA7* was significantly reduced in *in vitro* 4 day bradyzoites compared to that in the *in vitro* 2 day tachyzoites and then restored to near tachyzoite's levels in the *in vitro* 21 day bradyzoites (Buchholz et al., 2011). The expression of *GRA4, GRA6*, and *GRA8* has been reported to be reduced or even non-detectable in the bradyzoites (Ferguson, 2004). The different expression levels of some *GRAs* in different life cycle stages indicate that the roles played by *GRAs* may play parasite stage-dependent.

## Conclusion

GRAs play key roles in modulating host-parasite interactions, such as parasite vacuole remodeling, nutrient uptake and manipulation of host signaling pathways (Nadipuram et al., 2016). In this study, 17 Δ*GRA* mutant *T. gondii* strains were successfully generated using CRISPR-Cas9 technique. The role of these 17 *GRA* genes in the pathogenicity of *T. gondii* RH strain was investigated *in vitro* and *in vivo*. We report here that no significant difference was detected between Δ*GRA* knockouts and wild-type RH strains. These findings indicate that *GRA* genes examined in this study are not absolutely essential for *T. gondii* RH virulence, suggesting that other virulence factors may be involved in these processes or that virulence of *T. gondii* is the result of a multigene effect. We also investigated the patterns of gene expression and bioinformatics features of the 17 *GRAs*, by parasite cell cycle phases, life cycle stages and genotypes. Results indicated that the expression of *GRAs* can vary across life cycle stages or genotypes of *T. gondii*. Functional analysis of these *GRAs* in other parasite strains or life cycle forms is therefore of high importance and may further elucidate the pathogenic role of *GRAs* in *T. gondii* infection.

## Ethics statement

The study was approved by the Animal Administration and Ethics Committee of Lanzhou Veterinary Research Institute, Chinese Academy of Agricultural Science (Permit No. LVRIAEC-2017-006). All mice were handled humanely in strict accordance with the Guidelines and Animal Ethics Procedures of the People's Republic of China.

## Author contributions

X-QZ, J-LW, and HE designed the study and critically revised the manuscript. M-JB performed the experiments, analyzed data, and drafted the manuscript. Q-LL, KC, and L-BN participated in the implementation of the study. All the authors read and approved the final manuscript.

### Conflict of interest statement

The authors declare that the research was conducted in the absence of any commercial or financial relationships that could be construed as a potential conflict of interest.
