# Supplementary material for: Functional Characterization of Dense Granule Proteins in Toxoplasma gondii RH Strain Using CRISPR-Cas9 System
Source: Front Cell Infect Microbiol. 2018 Aug 28;8:300. doi: 10.3389/fcimb.2018.00300 (PMC6121064; doi:10.3389/fcimb.2018.00300)
Supplement: Supplementary file 1 [file Table_1.DOC]

**TABLE S1****｜The information of GRA genes, CRISPR sgRNAs and identification primers used in this study**

| **Name** | **sgRNA** | **KO-Forward primer** | **KO-Reverse primer** | **RT-Forward primer** | **RT-Reverse primer** |
| --- | --- | --- | --- | --- | --- |
| GRA11 | GCGTCGCACGAGACACCAGG | CTGGTCGCAGCGGTCATCTAC | TGATCCACCTGCTCGGTCTGT | CTGGTCGCAGCGGTCATCTAC | TGATCCACCTGCTCGGTCTGT |
| GRA12 bis | GGTACGGCAACCAACTGCCC | CTCTGCGGGTGCCTTGTGAGA | GATGAAGCCGGAGACGATGCTG | GATGTTCAACGCGGTGCG | AGACGATGCTGCCCTTCC |
| GRA13 | GCAGGAAAGTGCCTTCGTGA | CCACTCCGTTTGCTTCTGCTCG | CCAGGCTTTCCGTTGTACCTCCA | CCACTCCGTTTGCTTCTGCTCG | CCAGGCTTTCCGTTGTACCTCCA |
| GRA14 | GGACATCAATGGTCTCCATG | TGGAGTTCTTAGGCGTTTACCC | CAGCAACAACAGAGGCGACAT | TGGAGTTCTTAGGCGTTTACCC | CAGCAACAACAGAGGCGACAT |
| GRA20 | GTTGTCCAACAAGGAGGTGC | CGACAACGGAAACGGCACTAC | TGCACCCTCAACACTGACAGCAC | CGACAACGGAAACGGCACTAC | TGCACCCTCAACACTGACAGCAC |
| GRA21 | GCTGCACATGAGAAGGCGCA | TCACTGGCGGTAGGCAAATGG | AGCGGTGGCTCAAGGTCAAAA | TCACTGGCGGTAGGCAAATGG | AGCGGTGGCTCAAGGTCAAAA |
| GRA28 | GGCAAGCTTCCAGCAACCAG | CAATCCTCATTCTGCCCACCAC | CACCTCTTCGTTTCCGCCTTC | CAATCCTCATTCTGCCCACCAC | CACCTCTTCGTTTCCGCCTTC |
| GRA29 | GCTGGATCGGACGACAGGCG | CAAATCTTGGCTCTGCTTC | GTTCTTCACCTCGCTCTTC | CAAATCTTGGCTCTGCTTC | GTTCTTCACCTCGCTCTTC |
| GRA30 | GGCTCTGACTCCGCATGACG | TAAACCGCCCGAGAGAGCA | AGCAAACCCCGTCGAGACC | CTGCTACCGTGGACATCG | GGACCAACCGAAACAACA |
| GRA31 | GAGGGCCTTCTCGTGGAAGA | AAAGGAAACATCCCCGAAA | TGGAAGGACCTGGTAACAG | AAAGGAAACATCCCCGAAA | TGGAAGGACCTGGTAACAG |
| GRA33 | GCGCGAAGAGCGACATTCCT | TTCGGGAGAACTGGCAAAGGG | GGCGTCTGATGCAACGGACAA | TTCGGGAGAACTGGCAAAGGG | GGCGTCTGATGCAACGGACAA |
| GRA34 | GCGTTCAGGAAACGAAGCAG | ACACTGGTTGTCTGTTTCT | CTTGCCCCTTGTTATCTTC | ACACTGGTTGTCTGTTTCT | CTTGCCCCTTGTTATCTTC |
| GRA35 | GCTACCGCAGCAGCTGAAAT | AGAGTCTGAGGCCAAGGGT | GCTGCGTGTACAGGATGTG | AGAGTCTGAGGCCAAGGGT | GCTGCGTGTACAGGATGTG |
| GRA36 | GCTCCGACGTGAGATGGACA | GCCTTGTTGTCATTCTACG | CTCTCTCCACCTCCTCTTC | GCCTTGTTGTCATTCTACG | CTCTCTCCACCTCCTCTTC |
| GRA37 | GGAGAAGGAGAGGTTGACAC | ATCGCCTTTCTCGCCCTAA | CACGCTGTTGTTCATCCCT | ATCGCCTTTCTCGCCCTAA | CACGCTGTTGTTCATCCCT |
| GRA38 | GACTGTGAAACGAAATATGT | AGAGACACGACAGGAGGAC | CTACAAGAAGACAAGCAGA | AACAATCGGCGGATTCCA | TTCCTCAGCCCATAGGTC |
| GRA40 | GTATCTGCAGCTGCAGAGAG | AAGCCGTTTCCACTCCTGT | CTCCTCCCGTCTTTGTTCC | GCCCACGCCTATTCCAGC | ATGGGCACTGCGTGTTGC |
